# Intrathecal Therapies for Neurodegenerative Diseases: A Review of Current Approaches and the Urgent Need for Advanced Delivery Systems

**DOI:** 10.3390/biomedicines13092167

**Published:** 2025-09-05

**Authors:** Thomas Gabriel Schreiner, Manuel Menéndez-González, Oliver Daniel Schreiner, Romeo Cristian Ciobanu

**Affiliations:** 1 Department of Medical Specialties III, Faculty of Medicine, “Grigore T. Popa” University of Medicine and Pharmacy, 700115 Iasi, Romania; schreiner.thomasgabriel@yahoo.com (T.G.S.); oliver-daniel.schreiner@academic.tuiasi.ro (O.D.S.); 2 First Neurology Clinic, “N. Oblu” Clinical Emergency Hospital, 700309 Iasi, Romania; 3 Facultad de Medicina y Ciencias de la Salud, Universidad de Oviedo, Calle Julián Clavería s/n, 33006 Oviedo, Spain; menendezgmanuel@uniovi.es; 4 Department of Neurology, Hospital Universitario Central de Asturias, Avenida Roma s/n, 33011 Oviedo, Spain; 5 Spain Instituto de Investigación Sanitaria del Principado de Asturias, Avenida Roma s/n, 33011 Oviedo, Spain; 6 Medical Oncology Department, Regional Institute of Oncology, 700483 Iasi, Romania; 7 Department of Electrical Measurements and Materials, Gheorghe Asachi Technical University, 700050 Iasi, Romania

**Keywords:** neurodegenerative diseases, drug delivery systems, Alzheimer’s disease, Parkinson’s disease, Huntington’s disease, amyotrophic lateral sclerosis, clinical trials, antisense oligonucleotides, gene therapy, intrathecal pumps, pseudodelivery

## Abstract

Neurodegenerative diseases (NDDs) pose an immense global health burden, and developing effective treatments is hindered by the blood–brain barrier (BBB). Intrathecal (IT) administration of therapeutics directly into the cerebrospinal fluid (CSF) bypasses the BBB, offering a promising avenue for antisense oligonucleotides (ASOs), gene therapies, antibodies, and stem cells for these disorders. This review synthesizes the current landscape of IT therapies for Alzheimer’s disease, Parkinson’s disease, Huntington’s disease, and Amyotrophic Lateral Sclerosis based on the current literature and ClinicalTrials.gov. We highlight key trials and approaches, including the success of ASOs in spinal muscular atrophy and recent progress in other NDDs. However, the efficacy of these novel treatments is often constrained by the limitations of first-generation IT delivery systems, which struggle with uneven distribution, systemic leakage, and the demands of modern biologics. Drawing from recent analyses, we underscore the critical shortcomings of current devices and point out the innovations needed in shaping next-generation systems: subcutaneous access ports, CSF flow platforms, AI-driven adaptive dosing, nanoporous membranes, intrathecal pseudodelivery, and hydrogel scaffolds. We conclude by emphasizing the urgent need for these advanced IT drug delivery systems, alongside rigorous comparative assessments, cost–benefit analyses, and clear regulatory pathways to fully realize the potential of emerging CNS therapies and transform NDD management.

## 1. Introduction

Neurodegenerative diseases (NDDs) encompass a wide array of chronic, progressive disorders characterized by the gradual loss of neurons, and represent a growing burden for aging populations worldwide, posing significant challenges for healthcare systems due to their high morbidity, long disease course, and the lack of curative treatments [1]. This heterogeneous group of disorders comprises, among others, Alzheimer’s disease (AD), Parkinson’s disease (PD), Huntington’s disease (HD), and Amyotrophic Lateral Sclerosis (ALS). AD is the most common cause of dementia, affecting over 50 million individuals worldwide [2]. Clinically, it is characterized by progressive memory loss, cognitive dysfunction, and behavioral changes, primarily resulting from the accumulation of extracellular amyloid beta (Aβ) plaques and intracellular neurofibrillary tangles composed of hyperphosphorylated tau protein [3]. These aggregates disrupt synaptic function, trigger neuroinflammation, and promote neurodegeneration, starting in the hippocampus and ultimately affecting the entire cerebral cortex. PD, the second most frequent NDD, is a progressive movement disorder primarily resulting from the degeneration of dopaminergic neurons in the substantia nigra pars compacta [4]. Clinically, PD is characterized by motor symptoms, including tremors, bradykinesia, rigidity, and postural instability, as well as non-motor symptoms such as cognitive impairment, depression, and autonomic dysfunctions, which significantly impact the patient’s quality of life [5]. The pathological hallmarks of PD include the presence of Lewy bodies, composed of misfolded α-synuclein and extensive loss of dopaminergic neurons [6]. HD is a rare, autosomal dominant NDD caused by a CAG trinucleotide repeat expansion in the *HTT* gene, leading to the production of mutant huntingtin protein [7]. This toxic protein accumulates in neurons, causing progressive motor dysfunction, psychiatric symptoms, and cognitive decline [8]. Compared to the other NDDs, HD is unique in that the genetic cause is known and consistent across all affected individuals, making it a prime candidate for targeted molecular therapies. Finally, ALS is a rapidly progressive neurodegenerative disorder that affects both upper and lower motor neurons, leading to muscle weakness, atrophy, fasciculations, and ultimately respiratory failure [9]. Most cases are sporadic, but approximately 10% are familial, often linked to mutations in genes such as *SOD1*, *C9orf72*, and *TARDBP* [10].

Despite extensive research, treatment strategies for NDDs remain largely symptomatic, offering only modest improvements in quality of life and limited slowing of disease progression. The complexity of NDDs arises from the incompletely understood, diverse etiologies, as well as the co-existence of multifactorial pathogenic mechanisms, including protein misfolding and aggregation, mitochondrial dysfunction, oxidative stress, excitotoxicity, impaired axonal transport, and chronic neuroinflammation [11]. Moreover, the blood–brain barrier (BBB), the highly selective semipermeable border that protects the central nervous system (CNS), simultaneously restricts the entry of potentially therapeutic agents [12]. This has significantly hindered the effective delivery of emerging therapies, particularly those based on large or polar molecules such as antisense oligonucleotides (ASOs), monoclonal antibodies, viral vectors for gene therapy, and stem cells. With many promising agents failing to reach therapeutic concentrations in target CNS regions when administered systemically, it is currently a critical unmet need for delivery strategies that can bridge this gap.

In this context, intrathecal (IT) drug administration—the delivery of therapeutic agents directly into the cerebrospinal fluid (CSF)—addresses this need by bypassing the BBB and achieving immediate proximity to the CNS milieu [13]. This approach can yield markedly higher central drug concentrations with lower systemic exposure, enabling the use of modalities that would otherwise be pharmacologically inaccessible. In recent years, IT delivery has moved beyond niche indications and into the mainstream of experimental neurotherapeutics, underpinning trials of ASOs, gene therapy vectors, targeted immunotherapies, and regenerative cell-based products. Yet, the current literature remains fragmented: while individual studies demonstrate feasibility and preliminary efficacy, there is no comprehensive synthesis that critically appraises device technology, pharmacokinetics, and safety across NDDs. Addressing this knowledge gap is essential to accelerate translation, optimize delivery systems, and inform regulatory pathways for next-generation CNS therapies.

The aim of this review is to synthesize the current landscape of IT therapies for the aforementioned NDDs, based on the current literature and ClinicalTrials.gov. The most relevant trials and therapeutic approaches are highlighted, including the success of ASOs in Spinal muscular atrophy and recent progress in other NDDs. To offer a precise picture of the situation, the limitations of first-generation IT delivery systems are also presented, which struggle with uneven distribution, systemic leakage, and the demands of modern biologics. These critical shortcomings of current devices are detailed, along with the innovations shaping next-generation systems: subcutaneous access ports, CSF flow platforms, AI-driven adaptive dosing, nanoporous membranes, intrathecal pseudodelivery, and hydrogel scaffolds. The conclusion emphasizes the urgent need for the development of advanced IT drug delivery systems, alongside rigorous comparative assessments, cost–benefit analyses, and clear regulations to fully realize the potential of emerging CNS therapies, transform NDD management, and improve patients’ quality of life and lifespan.

## 2. Intrathecal Route: Principles and First-Generation Devices

The intrathecal (IT) route of drug administration involves delivering therapeutic agents directly into the CSF, bypassing the restrictive blood–brain barrier (BBB). This enables therapeutics, particularly large molecules such as biologics, ASOs, and stem cells, to reach target tissues in the CNS at therapeutic concentrations. Although the clinical application of IT delivery is still evolving, the great potential of this method depends primarily on understanding the anatomical and physiological principles that underlie the intrathecal route and the improvement in first-generation devices.

The relevant anatomical and physiological aspects from a therapeutic perspective are related to the meninges surrounding the CNS and the role of the CSF in providing mechanical cushioning, immunological protection, nutritional support, and waste exchange [14]. The CSF, summing approximately 150 mL, circulates through the ventricular system and subarachnoid space, undergoing complete turnover approximately four to five times per day via absorption at the arachnoid villi into the venous sinuses [15]. Moreover, when considering peripherally administered medication, the BBB acts as a highly selective barrier for substances entering the CNS from the systemic circulation [16]. Although this structure plays a crucial role in protecting the brain from toxins and maintaining homeostasis, it severely restricts the entry of potentially therapeutic macromolecules.

Intrathecal administration bypasses the BBB by directly accessing the CSF. From the CSF, therapeutic agents can distribute along the neuraxis, particularly targeting the periventricular, cortical, and spinal cord regions. IT administration follows distinct pharmacokinetic principles compared to systemic delivery [17]. Upon injection into the lumbar subarachnoid space, therapeutics distribute in a rostral direction due to pulsatile CSF flow driven by cardiac and respiratory cycles [18]. The distribution and clearance of drugs within the CSF are influenced by several factors, including drug-related factors such as molecular size, charge, or lipophilicity, as well as patient-related factors, including CSF flow dynamics [19]. Larger molecules (e.g., ASOs, proteins, viral vectors) exhibit limited diffusion and are more reliant on convective CSF flow. Charged and hydrophilic molecules tend to remain within the CSF compartment, whereas lipophilic compounds have an increased biodisponibility [20]. Factors related to administration protocols, such as injection volume, drug concentration, and excipients, can also affect CNS spread and penetration. When considering CSF dynamics, pathological conditions such as hydrocephalus or spinal stenosis can alter mechanically drug dispersion and lead to localized drug accumulation or reduced efficacy [21]. Clearance of IT-administered drugs occurs primarily via bulk flow into systemic circulation through arachnoid granulations, glymphatic drainage pathways, and, to a lesser extent, perivascular routes [22]. Or, in NDDs, particularly AD, there is a reduced rate of substance clearance from the CNS, influencing the drug concentration balance [23]. The half-life of IT-delivered substances in the CSF is typically longer than plasma administered drugs, enhancing the potential for sustained CNS exposure with appropriately designed delivery systems.

From a historical perspective, the concept of IT administration dates back to the early 20th century, when it was originally employed for spinal anesthesia and the treatment of infectious meningitis [24]. The use of the IT route for chronic neurological conditions, however, gained importance in the latter half of the 20th century. Among the key milestones worth mentioning are the 1970s and 1980s, when implantable pumps for the continuous IT administration of analgesics (e.g., morphine) and muscle relaxants (e.g., baclofen) were developed [25]. During the 1990s and 2000s, IT delivery was proposed for chemotherapy in CNS malignancies and leptomeningeal metastases [26]. In 2016, the Food and Drug Administration (FDA) approved Nusinersen, an ASOs administered via intrathecal injection for spinal muscular atrophy (SMA), marking the first intrathecal gene-targeted therapy in humans for broad clinical use [27]. More recently, in 2023, the FDA approved tofersen, an intrathecally administered ASO for *SOD1*-mutant ALS patients [28]. These milestones are a clear indication of the potential of IT routes in treating various NDDs, with the guidelines and recommendations expected to broaden in the next few years.

The success of IT therapies depends not only on the therapeutic molecule, which is expected to be improved thanks to the advancements in drug design, but also heavily relies on the reliability, safety, and reproducibility of the delivery system. First-generation intrathecal devices include spinal needles for repeated lumbar punctures, percutaneous catheters, and implantable infusion systems [29]. While each method has specific clinical applications and its particularities, they collectively represent the foundational tools for CNS-directed therapy.

For many IT-delivered biologics and ASOs, including nusinersen and tofersen, drug administration is currently achieved via lumbar puncture using a spinal needle. This technique, involving the percutaneous insertion of a needle (typically 20–25 gauge) between the L3 and L5 vertebrae to access the subarachnoid space, is preferred due to its simplicity and regulatory acceptance [30]. The advantages of this technique worth mentioning are the low initial costs, minimal invasiveness, and the absence of surgical implantation requirements. Still, procedural discomfort and anxiety, particularly challenging in patients with scoliosis, spinal degenerative disorders, or obesity, the risk of post-puncture headache, and the impracticality for frequent or long-term use are the most relevant limitations of this approach [31]. Additionally, there are factors such as steep rostrocaudal gradients after lumbar bolus injections and substantial sensitivity to injection volume, rate, and patient-specific CSF flow that limit reliable supratentorial/parenchymal exposure for large biologics (e.g., antibodies, ASOs) and make dosing less reproducible across subjects. Recent in vitro and in silico human models confirm that modifying parameters (volume, rate, injection site) can improve cranial transport, and even optimized bolus protocols usually give more heterogeneous brain exposure than continuous approaches [32].

For more frequent or continuous delivery, intrathecal catheters may be employed. These catheters are surgically placed into the lumbar or thoracic subarachnoid space and are often connected to an external port or reservoir. In daily clinical practice, they are currently used for the administration of intrathecal baclofen in cases of severe spasticity [33], when orally administered medication is insufficient, and for the delivery of chemotherapeutics in CNS tumors [34]. In the research field, they are used for experimental gene or cell therapy studies requiring repeated access. The most relevant challenges faced by clinicians in this procedure are the risk of infection and catheter dislodgement, the potential for fibrosis and catheter occlusion, and the necessity of routine monitoring and maintenance [35]. Although more invasive than the classical lumbar puncture, intrathecal catheters allow for controlled delivery, programmable dosing, and the possibility of combination therapies.

Finally, implantable intrathecal infusion pumps provide programmable, long-term delivery of IT medications and are particularly useful in managing chronic pain and spasticity [36]. Devices such as the Medtronic SynchroMed™ II system (Medtronic, Medtronic Parkway Minneapolis, MN, USA) consist of a subcutaneously implanted reservoir connected to an intrathecal catheter. The primary strengths of this method lie in its continuous steady-state drug delivery and the reduced need for frequent lumbar punctures, which is particularly convenient for patients with improved compliance [37]. Still, the approach requires surgical implantation, peri- and postprocedural risks such as pump malfunction, infections, and catheter-related complications [38]. While currently used primarily for small-molecule therapeutics, there is limited experience with large biomolecules (e.g., ASOs, viral vectors). Still, there is growing interest in adapting these systems for the delivery of biologics and gene therapies; however, compatibility and stability issues should be addressed, particularly when considering patients with NDD who require long-term follow-up.

Indwelling catheters with subcutaneous access ports and implantable pumps enable repeated bolus dosing, leading to improved adherence and cumulative exposure; however, they also carry risks of device-related infections and catheter malfunctions. Contemporary series report perioperative or device-related infection rates for intrathecal drug delivery systems in the 2–6% range, with long-term series indicating roughly 0.7% per patient-year in some cohorts. Programmable implantable pumps (continuous infusion) typically provide the steadiest CSF levels and, in preclinical and clinical examples, can produce more homogeneous CNS exposure for large molecules than isolated lumbar boluses, a potential pharmacokinetic advantage for large biologics or enzyme/gene therapies where sustained gradients improve parenchymal penetration.

**Figure 1:**
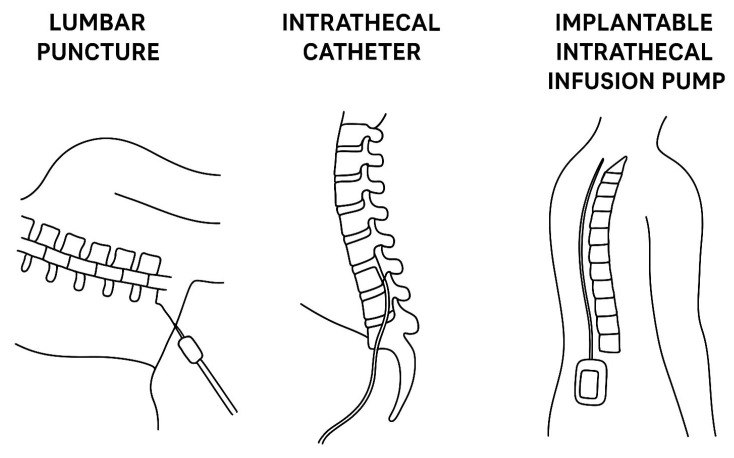
Comparative illustration of the most frequent approaches for intrathecal therapy delivery (lumbar puncture versus intrathecal catheter versus implantable intrathecal infusion pump).

Table 1 summarizes the most relevant first-generation devices and common approaches for IT drug delivery, while Figure 1 reveals in a schematic manner the differences between the three approaches.

## 3. Intrathecal Therapies in NDDs: A Clinical Trial Landscape

We have structured this chapter in a systematic manner to cover the most significant clinical trials conducted in recent years, with a focus on the most relevant NDDs.

### 3.1. Alzheimer’s Disease

While AD remains the most prolific among NDDs in terms of clinical trials of novel therapeutic compounds [39], an incompletely explored part is related to IT-delivered anti-AD drugs. Based on the current literature and ClinicalTrials.gov, three main classes of pharmaceutics are relevant: ASOs targeting Tau protein and Amyloid Precursor Protein (APP), anti-amyloid beta (Aβ) antibodies, and gene therapies.

#### 3.1.1. Asos in Alzheimer’s Disease

The development of ASOs that modulate RNA expression represents a transformative approach in the treatment of NDDs. In AD, ASOs target either tau pathology, with BIIB080 as a representative compound, or APP. Hyperphosphorylated tau remains one of the main pathological changes, strongly correlated with AD’s clinical progression, making it an attractive target for ASO therapy [40]. BIIB080 (also known as IONIS-MAPTRx) is a tau-targeted ASO developed through a collaboration between Ionis Pharmaceuticals and Biogen [41]. Administered intrathecally, BIIB080 binds to the pre-mRNA of the *MAPT* gene, promoting its degradation via RNase H-mediated cleavage, thereby reducing tau protein synthesis. Initial clinical evaluation of BIIB080 was the object of a Phase 1b randomized placebo-controlled trial (NCT03186989) [42]. This dose-escalation study enrolled patients with mild AD and demonstrated favorable safety and tolerability of up to 60 mg IT every 12 weeks. Notably, CSF total tau and phosphorylated tau levels decreased in a dose-dependent manner, with reductions sustained for several months post-injection. These findings were corroborated by exploratory neuroimaging endpoints, which suggested potential stabilization of disease-related atrophy patterns. The trial’s results laid the groundwork for a larger Phase 2 study (NCT04986707), which is currently enrolling participants to assess long-term efficacy and cognitive outcomes [43].

Although less developed than tau-targeted approaches, antisense oligonucleotides (ASOs) directed at APP mRNA represent an emerging strategy for reducing Aβ production, as Aβ is a key component of senile plaques. Preclinical models have demonstrated that APP-lowering ASOs can lead to substantial reductions in Aβ42 without perturbing essential physiological functions [44]. While no APP-targeted ASO has yet entered late-stage clinical trials for AD, early phase programs are in development, leveraging the IT route for precise CNS delivery. These programs aim to validate the concept of upstream mRNA intervention, potentially synergizing with or replacing anti-Aβ monoclonal antibodies [45]. Still, several challenges remain in the application of ASOs in AD management. Widespread tau expression requires regionally broad CNS distribution, which is currently limited by CSF flow heterogeneity. Dosing schedules involving IT injections every 12–16 weeks may present barriers to compliance in an elderly population [46]. Nevertheless, the preliminary promising data from BIIB080 clinical trials suggest that IT ASO therapy is biologically active and well-tolerated in AD, meriting continued investigation.

#### 3.1.2. Monoclonal Antibodies in Alzheimer’s Disease

Monoclonal antibodies targeting Aβ (e.g., aducanumab, lecanemab) have demonstrated modest clinical benefits in early-stage AD when administered intravenously [47]. However, systemic delivery often results in limited CNS penetration, with only ~0.1–0.5% of the injected dose crossing the BBB [48]. This inefficiency not only increases the required dose and costs but also contributes to peripheral adverse effects, such as amyloid-related imaging abnormalities (ARIA) [49]. A potential strategy to overcome these limitations could be the IT administration of anti-Aβ antibodies. By delivering antibodies directly into the CSF, higher CNS concentrations may be achieved with lower total doses, potentially enhancing efficacy while reducing systemic exposure. One of the first IT antibody trials in AD is NCT03397506, a Phase 1 study evaluating the safety and pharmacokinetics of an investigational anti-Aβ monoclonal antibody administered via the intrathecal route [50]. This open-label, dose-escalation study enrolled subjects with early symptomatic AD and examined various dosing regimens to determine optimal CNS bioavailability. Although detailed results have not yet been published, preliminary data suggest that IT delivery results in significantly higher CSF-to-plasma concentration ratios compared to IV administration, confirming the pharmacological rationale of this approach. With many unknowns still remaining, future trials are expected to assess not only pharmacokinetics but also target engagement (e.g., CSF Aβ reduction, PET imaging) and cognitive outcomes. Even this medication might raise challenges and have associated risks. For example, CSF dynamics and antibody distribution can have individual variability, complicating dosing standardization and requiring tailored therapies. Immunogenicity and ARIA risk remain concerns; however, initial findings suggest that intrathecal administration, by avoiding peripheral accumulation, may reduce the incidence of ARIA.

#### 3.1.3. Gene Therapy in Alzheimer’s Disease

Finally, an interesting and promising therapeutic avenue in NDDs comprises gene therapy, offering the potential to provide sustained, and in some cases, one-time disease-modifying interventions. Gene therapy in AD aims to either enhance neuroprotection or modulate pathogenic processes, such as amyloidogenesis and tau hyperphosphorylation [51]. The IT route provides a compelling delivery platform for gene therapies, offering direct access to the CNS, particularly for vector-based approaches. Historically, gene therapy in AD focused on targeted intracerebral delivery of neurotrophic factors such as nerve growth factor (NGF) [52] or brain-derived neurotrophic factor (BDNF) [53], based on the observation that basal forebrain cholinergic neurons are particularly vulnerable to degeneration in early AD. However, intracerebral injection is invasive, has limited distribution, and is logistically challenging. The IT route presents an alternative that is both less invasive and potentially capable of achieving more widespread CNS gene expression, particularly when using engineered viral vectors with enhanced neuronal tropism. Among the various vector platforms, adeno-associated virus (AAV) has emerged as the most widely used for CNS-directed gene therapy, due to its favorable safety profile, low immunogenicity, and capacity for long-term gene expression [54]. Specific serotypes, particularly AAV9 and AAVrh10, have demonstrated robust CNS tropism when administered via the IT route [55]. AAV9 has been shown to efficiently transduce neurons, astrocytes, and oligodendrocytes throughout the spinal cord and brain, especially in younger individuals and animal models [56]. AAVrh10, derived from non-human primates, offers similar advantages and may be less susceptible to neutralizing antibodies in the general population [57]. Preclinical studies have demonstrated that IT administration of these vectors can result in widespread CNS gene expression, particularly when administered at the lumbar cistern or via cisterna magna injection. The biodistribution achieved with IT delivery has been shown to encompass hippocampal, cortical, and thalamic regions—areas of high relevance in AD pathology.

The primary therapeutic strategies under investigation for IT gene therapy in AD can be categorized into three main areas: neurotrophic support, modulation of amyloid and tau, and support for synaptic and mitochondrial function [58]. With early-stage AD being characterized by a loss of cholinergic neurons in the basal forebrain, neurotrophic factors such as NGF and BDNF have been investigated for their potential to support these neurons. In early clinical studies (e.g., NCT00087789), NGF gene delivery via stereotactic injections has demonstrated long-term neuronal viability, as evidenced by the upregulation of choline acetyltransferase (ChAT) and persistent transgene expression for several years [59]. However, the limited distribution and surgical risk prompted the exploration of IT delivery as a less invasive, scalable approach. Preclinical IT-AAV-NGF studies have demonstrated successful transgene expression in hippocampal and cortical neurons, accompanied by improvements in memory tasks.

Another promising application is the delivery of genes that either suppress amyloidogenic pathways or promote tau clearance. Preclinical models have utilized AAV-mediated delivery of neprilysin, an enzyme that degrades Aβ, resulting in a reduced plaque burden and improved cognition [60]. Similarly, AAV vectors encoding tau-targeting intrabodies or microRNAs have shown efficacy in reducing tau pathology when delivered via the CSF [61]. More recent strategies aim to enhance synaptic plasticity and metabolic resilience in AD neurons. PGC-1α, a regulator of mitochondrial biogenesis, has been delivered via AAV to improve neuronal energy metabolism and reduce oxidative stress [62]. Similarly, overexpression of synaptic regulators such as PSD95 or Homer1a has been proposed to counteract synaptic failure; however, these remain primarily in early development [63].

While many IT gene therapy strategies for AD are in preclinical or early translational stages, several clinical trials and IND-enabling programs are underway. For example, a Phase 1/2 program (not yet publicly registered) is reportedly investigating IT-AAV-BDNF delivery in early-stage AD patients, building on animal data showing improved memory and synaptogenesis.

**Figure 2:**
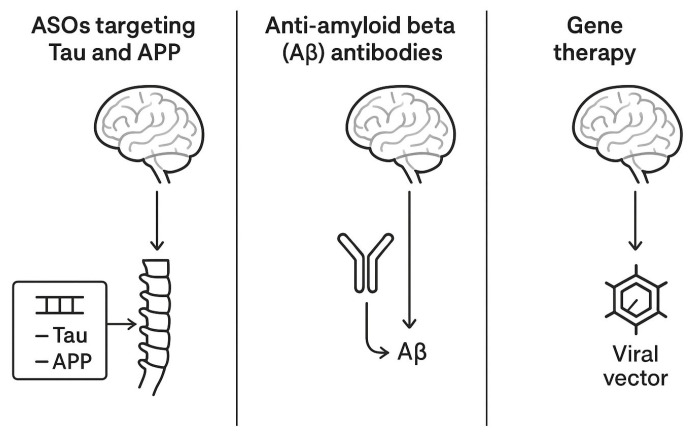
A comparative schematic representation of intrathecal therapies for AD.

Other industry-sponsored programs are targeting tau expression modulation using gene therapy vectors encoding tau-lowering RNA interference sequences, administered intrathecally for maximal parenchymal access. Table 2 summarizes the most relevant aspects of the clinical trial landscape in AD, while Figure 2 offers an illustrative comparative overview of the therapies.

Although the Gene Therapy Advisory Committee (GTAC) and regulatory bodies have raised concerns about vector immunogenicity, long-term expression control, and off-target effects, advances in self-limiting vectors, inducible promoters, and immunosuppression regimens are helping mitigate these risks [64]. Despite the promise, several challenges limit the near-term translation of IT gene therapy in AD:

- Nonuniform Vector Distribution: Even with intrathecal delivery, complete cortical and hippocampal coverage remains difficult, especially in older patients with altered CSF dynamics or brain atrophy.
- Preexisting Immunity to AAV Serotypes: Up to 60% of the population harbors neutralizing antibodies to common AAVs, which may reduce efficacy or pose safety risks.
- Dose Escalation Risks: High-dose IT vector administration, as seen in spinal muscular atrophy (SMA) gene therapy, carries risks of aseptic meningitis, liver toxicity, and sensory neuronopathy [65].

The coming years will be critical in determining whether gene therapy, along with the other aforementioned medication administered intrathecally, can transition from theoretical promise to daily clinical practice, improving the prognosis of AD patients.

### 3.2. Parkinson’s Disease

#### 3.2.1. Gene Therapy in Parkinson’s Disease

When considering PD, although current pharmacotherapies are more diverse than anti-AD medications and provide satisfactory motor symptom relief in most patients, they do not modify disease progression [66]. Furthermore, their efficacy diminishes over time, with late-stage PD patients suffering from motor fluctuations, dyskinesias, and the exacerbation of non-motor symptoms [67]. Consequently, disease-modifying therapies that target the underlying neurodegenerative processes are needed. Gene therapy and neurotrophic factor delivery have emerged as promising strategies, particularly when administered via intrathecal or cisternal routes, having advantages over the peripheral delivery method. This section primarily focuses on gene therapy targeting *GBA1* mutations, and neurotrophic factor delivery, highlighting both their mechanistic rationale and the translational challenges associated with them.

Mutations in the *GBA1* gene, which encodes the lysosomal enzyme glucocerebrosidase (GCase), represent the most common genetic risk factor for PD [68]. Both homozygous and heterozygous GBA1 mutations reduce GCase activity, resulting in impaired lysosomal function, accumulation of α-synuclein, and increased neuronal vulnerability [69]. *GBA1* mutation carriers tend to have earlier PD onset, faster progression, and more severe non-motor symptoms, particularly cognitive impairment [70]. Enhancing GCase activity has thus become a therapeutic strategy aimed at restoring lysosomal homeostasis, improving α-synuclein clearance, and slowing disease progression [71]. Small-molecule chaperones and enzyme replacement therapies have been tested with limited success due to insufficient CNS penetration. In contrast, AAV-mediated gene therapy provides a means to deliver the *GBA1* gene directly to affected neurons, thereby enabling sustained enzymatic activity following a single administration.

PR001 is an investigational AAV9-based gene therapy that delivers a functional copy of the human *GBA1* gene under a CAG promoter [72]. Developed by Prevail Therapeutics (now part of Eli Lilly), PR001 is designed to increase GCase expression throughout the CNS, following a single intrathecal administration into the cisterna magna. This delivery site, located at the cranial end of the spinal subarachnoid space, is advantageous for distributing viral vectors to both the brain and spinal cord through CSF circulation, with increased access to midbrain structures such as the substantia nigra. Preclinical studies in rodent and non-human primate models of *GBA1*-associated PD demonstrated that cisterna magna injection of PR001 leads to widespread vector biodistribution, robust transgene expression in cortical and subcortical regions, increased GCase activity and reductions in α-synuclein accumulation. Behavioral improvements and neuroprotection were also observed in these models, supporting the translational potential of this approach [73].

PR001 is currently under investigation in a Phase 1/2 clinical trial (PROPEL; NCT04127578) in patients with PD and biallelic or heterozygous *GBA1* mutations. The trial is an open-label, dose-escalation study evaluating safety, tolerability, and exploratory efficacy of a one-time cisterna magna infusion of PR001. Early data from this trial have shown an acceptable safety profile at low and intermediate doses, with transient fever and headache as the most common adverse events, with dose-dependent increases in CSF GCase activity, sustained for several months post-administration. Preliminary biomarker signals suggested a reduction in CSF α-synuclein levels. Still, longer-term follow-up and efficacy data, including motor and cognitive assessments, are pending. A parallel natural history study (NCT04128245) is also being conducted to better understand disease progression in *GBA1*-PD and facilitate the interpretation of endpoints [74].

Several challenges and aspects should be addressed to improve the outcomes of this therapy. Firstly, patient selection should be more rigorous. While *GBA1* mutations are a logical starting point, their clinical heterogeneity necessitates precise stratification. Further studies are needed to evaluate whether non-*GBA1* PD patients may benefit from similar lysosomal-targeted therapies [75]. The risk of AAV-neutralizing antibodies and cellular immune responses still remains; therefore, pre-screening for anti-AAV9 titers and optimized immunosuppression protocols are essential [76]. Finally, vector engineering to enhance cell-specific tropism and optimize spread to the CSF and CNS remains a priority.

#### 3.2.2. Neurotrofic Factors in Parkinson’s Disease

**Figure 3:**
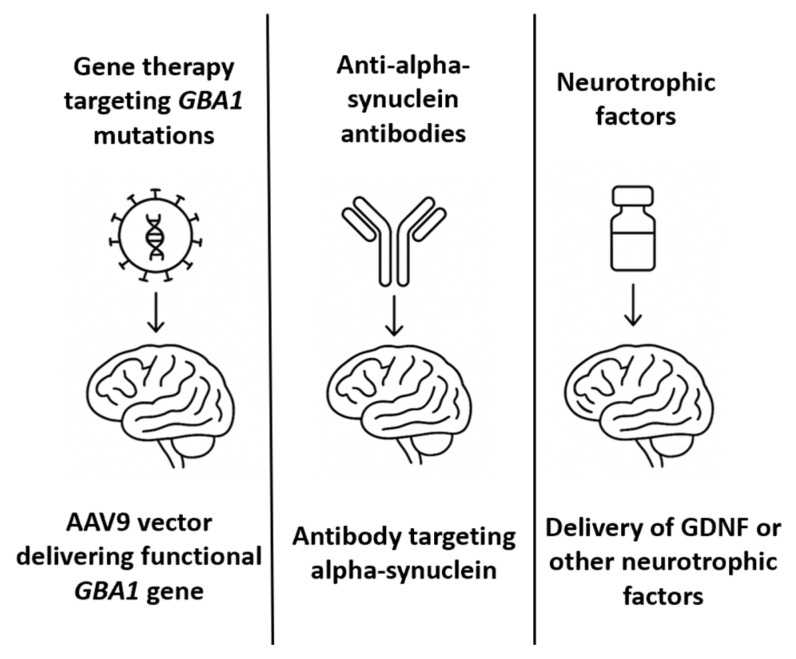
A comparative schematic representation of intrathecal therapies for Parkinson’s disease.

A long-standing therapeutic hypothesis in PD is that the exogenous administration of neurotrophic factors could support the survival of dopaminergic neurons, promote regeneration, and slow disease progression [77]. Among the most studied is glial cell line-derived neurotrophic factor (GDNF), which binds to the GFRα1/RET receptor complex to activate intracellular survival pathways in nigrostriatal neurons [78]. In PD patients, endogenous GDNF expression is significantly reduced in the substantia nigra [79]. Preclinical studies in 6-OHDA and MPTP models have demonstrated that GDNF delivery can reverse dopaminergic degeneration and restore motor function. Initial relevant clinical trials of GDNF in PD include an open-label Phase 1 study in which patients received continuous intraputaminal GDNF infusion, resulting in improved motor scores and PET-documented dopaminergic activity [80]. A subsequent randomized, placebo-controlled Phase 2 trial failed to meet primary endpoints, raising questions about efficacy, placebo response, and delivery variability. Considering the limitations associated with intracerebral infusion, alternative delivery routes—including intrathecal and intracisternal administration—have been explored to achieve broader CNS distribution. Preclinical studies have demonstrated that GDNF delivered intrathecally or via the cisterna magna can diffuse through perivascular spaces and reach midbrain targets; however, the concentrations achieved in the putamen are lower than those achieved via direct infusion [81]. To overcome these limitations, gene therapy vectors encoding GDNF have been developed. AAV2-GDNF was tested in Phase 1/2 trials via stereotactic injection into the putamen [82]. These trials demonstrated safety and target expression but were limited by the slow onset of clinical benefit and concerns about retrograde transport to the substantia nigra. Alternatively, several biotechnology companies and academic groups are now investigating next-generation neurotrophic factor therapies, including encapsulated cell therapies (e.g., NTCELL), which secrete GDNF locally after implantation into the striatum, synthetic mimetics of GDNF that are small enough to cross the BBB and bind GFRα1/RET receptors and mRNA therapies encoding neurotrophic factors [83]. Table 3 summarizes the most relevant milestones in PD therapeutic approaches, while Figure 3 offers an illustrative comparative overview of these therapies.

It is clear that future strategies must integrate advanced delivery systems, such as convection-enhanced delivery (CED), nanoparticle carriers, or gene therapy vectors optimized for IT use. Biomarker-driven patient selection and real-time imaging of target engagement will be critical for trial success.

### 3.3. Huntington’s Disease

In the context of HD, ASOs are used to selectively degrade *HTT* mRNA, thereby reducing production of both mutants and, in some cases, wild-type huntingtin proteins [84]. Preclinical studies in HD mouse and non-human primate models have demonstrated that ASOs-mediated *HTT* suppression results in significant reductions in m*HTT* levels in the brain and CSF, restoration of transcriptional dysregulation, and improvements in motor performance and survival [85]. The pioneering molecule in this domain is Tominersen (formerly IONIS-HTTRx or RG6042), a fully phosphorothioate-modified 2′-O-methoxyethyl ASO developed by Ionis Pharmaceuticals and Roche, targeting both wild-type and mutant *HTT* mRNA transcripts [86]. It was the first *HTT*-lowering therapy to enter human trials and remains the most extensively studied ASOs in HD. to date. The phase 1/2a Trial (NCT02519036) was the first-in-human study demonstrating that monthly IT administration of tominersen led to dose-dependent reductions (up to 40%) of m*HTT* in CSF in early manifest HD patients [87]. The drug was well tolerated at lower doses, and exploratory cognitive and motor endpoints trended positively. A phase 3 randomized, placebo-controlled trial (GENERATION HD1 trial–NCT03761849) aimed to evaluate the efficacy of tominersen in 791 early manifest HD patients [88]. Participants received either 120 mg every 8 weeks or every 16 weeks via lumbar puncture. However, in March 2021, the study was halted early due to a lack of efficacy and worsening outcomes in the higher-frequency dosing arm. The data showed modest reductions in *mHTT*, but the every-8-weeks group experienced more adverse events, including ventricular enlargement, increased neurofilament light chain (NfL) levels, and clinical deterioration [89]. These findings prompted a need for reassessment of *HTT*-lowering strategies. Still, post hoc analyses suggested that certain subgroups (e.g., younger patients with lower disease burden) may still benefit from therapy. Currently, a new trial (GENERATION HD2, NCT05686551) is ongoing, with tominersen being administered in lower and less frequent doses to reduce toxicity and maintain target engagement [90].

Given the potential risks of lowering wild-type *HTT*, another interesting alternative approach is based on allele-selective ASOs that preferentially target mutant *HTT* mRNA. These designs exploit single-nucleotide polymorphisms (SNPs) in linkage disequilibrium with the CAG expansion to selectively degrade the mutant transcript [91]. Programs such as Wave Life Sciences’ WVE-120101 and WVE-120102, which targeted specific SNPs (rs362307 and rs362331), initially showed suboptimal efficacy and were discontinued [92]. However, newer backbones and improved chemistry (e.g., PN backbone) have revived interest in next-generation SNP-targeting ASOs. The WVE-003 program (NCT05032196) is currently under evaluation for safety, tolerability, and target engagement after IT administration [93].

Unlike ASOs, gene therapy approaches aim to permanently suppress or repair the *HTT* gene using viral vectors such as adeno-associated viruses (AAVs). These vectors can deliver short-hairpin RNAs (shRNAs), artificial microRNAs (miRNAs), or zinc finger repressors directly into the brain, leading to long-lasting mHTT knockdown [94]. However, the large size of the *HTT* gene (exceeding AAV capacity) and broad CNS involvement of HD make gene therapy delivery particularly challenging. Consequently, many programs have opted for region-specific intracranial delivery, especially targeting the striatum and thalamus. One example is the AMT-130 (NCT04120493) trial. Developed by uniQure, this AAV5-based gene therapy expresses an miRNA targeting exon 1 of the *HTT* transcript. Administered via bilateral intrastriatal infusion using convection-enhanced delivery (CED), AMT-130 has shown sustained m*HTT* knockdown in preclinical models [95]. An ongoing Phase 1/2 trial is assessing the long-term safety, reduction in m*HTT* in CSF, and imaging-based biomarkers. Voyager Therapeutics’ VY-HTT01 (now discontinued) utilized an AAV vector with an artificial miRNA delivered directly into the striatum [96]. Despite promising preclinical data, the program was halted due to concerns over delivery complexity and safety. Currently, no AAV-based *HTT*-lowering therapy has utilized the intrathecal route in clinical trials, primarily due to limitations in vector spread and tropism. However, advances in capsid engineering (e.g., AAV.PHP.B, AAV9 variants) and promoters may enable IT or cisternal delivery of HTT-targeting vectors in future iterations [97].

**Figure 4:**
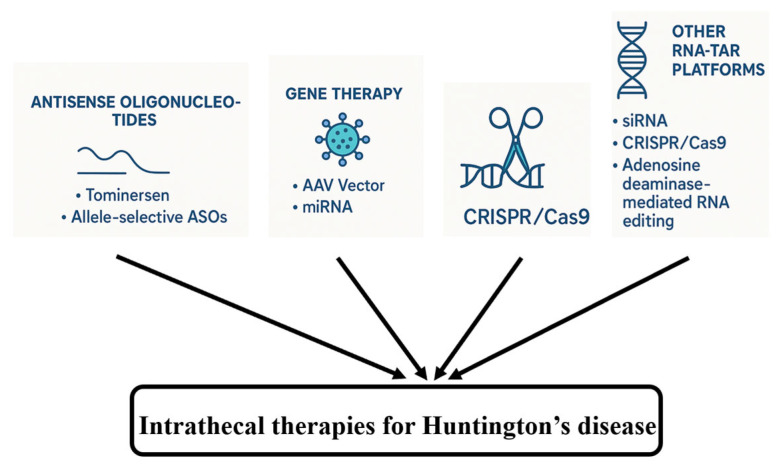
Intrathecal therapies for HD–an overview.

Beyond gene transfer, other RNA-targeted platforms are being explored for IT delivery. For example, similar to ASOs, small interfering RNAs (siRNAs) have been investigated for *HTT* suppression, though challenges related to stability and delivery persist. Preclinical studies have explored the use of CRISPR/Cas9 or CRISPR interference (CRISPRi) to silence or correct the *HTT* gene, but the delivery of large Cas9 constructs and potential adverse effects remain significant barriers [98]. Adenosine deaminase-mediated RNA editing offers a theoretical method to correct CAG expansions or disrupt m*HTT* translation. These techniques, summarized in Figure 4, are in early stages but may eventually complement IT delivery strategies.

### 3.4. Amyotrophic Lateral Sclerosis

Recent therapeutic advances, particularly in ASOs and gene therapy, offer new hope in the therapeutic landscape of ALS. IT delivery is central to their clinical deployment. ASOs can target several genes. One common target is *SOD1*, as *SOD1* mutations are among the most frequent genetic causes of ALS, accounting for ~20% of familial ALS cases and ~2% of all ALS [99]. Tofersen (BIIB067) is a phosphorothioate-modified 2′-O-methoxyethyl (2′-MOE) ASO developed to selectively degrade *SOD1* mRNA, thereby reducing toxic protein accumulation [100]. The Phase 1/2 Trial (NCT02623699) consisted of the ascending-dose administration of monthly IT injections of tofersen, which were generally well tolerated and resulted in dose-dependent reductions in CSF *SOD1* and neurofilament light chain (NfL) [101]. Subsequently, in the phase 3 VALOR Trial (NCT02623699), a double-blind, placebo-controlled trial involving 108 participants with confirmed *SOD1* mutations, tofersen failed to reach the primary endpoint. However, significant reductions in plasma NfL and CSF *SOD1* were observed, and exploratory analyses suggested a potential long-term benefit in early treated patients [102]. In April 2023, the FDA granted accelerated approval for tofersen based on biomarker evidence of target engagement, marking the first approved therapy targeting a genetic form of ALS [28].

Another interesting target for ASOs is *C9orf72*, particularly the hexanucleotide (GGGGCC) repeat expansions in *C9orf72*, which are present in ~40% of familial ALS cases and 5–10% of sporadic ALS cases [103]. ASOs targeting *C9orf72* mRNA or intronic repeat-containing RNAs are in active development. For example, BIIB078 (Ionis) was designed to selectively suppress the mutant *C9orf72* allele. Despite strong preclinical rationale, a Phase 1 trial (NCT03626012) was discontinued after failing to demonstrate clinical benefit or favorable biomarker profiles [104]. Wave Life Sciences (WVE-004), targeting repeat-containing transcripts, is currently being evaluated in a Phase 1b/2a trial (FOCUS-C9, NCT04931862). Preliminary results show a dose-dependent reduction in poly(GP) DPRs in CSF, suggesting pharmacodynamic engagement, though clinical data are pending [105].

Finally, mutations in FUS, an RNA-binding protein, are implicated in juvenile-onset ALS and drive toxic cytoplasmic mislocalization of the protein [106]. A preclinical ASO program by Ionis and Biogen targeting FUS mRNA has entered IND-enabling studies, with IT delivery planned based on animal efficacy in spinal motor neurons.

**Figure 5:**
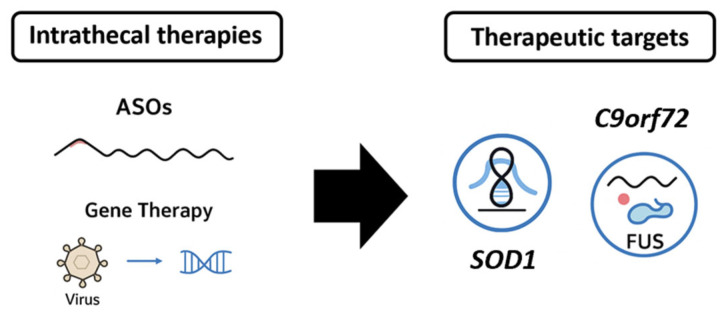
A schematic overview of intrathecal therapies and the main therapeutic targets for ALS.

In contrast to ASOs, gene therapy aims for more durable, potentially permanent, disease modification. In ALS, AAV vectors, particularly AAV9 and AAVrh10, are suitable for IT delivery. APB-102 is an AAVrh10 vector delivering an artificial microRNA targeting *SOD1* mRNA [107]. It is administered via intrathecal injection to ensure widespread spinal cord delivery. A Phase 1/2 trial (NCT04833907) is ongoing to assess safety, tolerability, and target engagement. Preclinical studies demonstrated the efficient knockdown of *SOD1* and preservation of motor function in murine models [108]. AAV9, the vector backbone for Zolgensma (approved for spinal muscular atrophy), is being adapted for ALS due to its demonstrated ability to cross the BBB when delivered systemically or via IT injection [109]. Passage Bio’s PBFT02, targeting GRN in frontotemporal dementia with TDP-43 pathology, utilizes cisterna magna administration—a route that may also benefit ALS gene therapies by providing superior CNS distribution [110]. TSHA-102 (Taysha Gene Therapies), a self-regulating gene therapy for Rett syndrome, is testing intrathecal dosing for sustained expression [111]. Similar regulatory switches could be used in future ALS constructs to fine-tune gene expression and reduce toxicity. Figure 5 summarizes, in an illustrative manner, the therapeutic directions in ALS.

### 3.5. Spinal Muscular Atrophy (SMA)—A Success Story and Path Forward

SMA, although not a classical NDD, is important to consider when evaluating IT-administered therapies from several perspectives. This devastating autosomal recessive neuromuscular disorder is caused by bi allelic deletions or mutations in the survival motor neuron 1 (*SMN1*) gene [112]. The resultant deficiency in the SMN protein leads to progressive degeneration of anterior horn cells in the spinal cord, culminating in muscular weakness, atrophy, and in severe cases, early mortality [113]. Historically, SMA has been one of the leading genetic causes of infant death, and until 2016, SMA’s management was purely supportive [114].

The therapeutic revolution began with the approval of nusinersen, followed by onasemnogene abeparvovec (gene therapy) and risdiplam (oral splicing modulator). Among these, nusinersen has been a landmark, administered directly into the CSF via lumbar puncture [115]. As an ASOs, nusinersen modulates *SMN2* pre-mRNA splicing to promote exon 7 inclusion, increasing functional SMN protein expression predominantly in the CNS [115]. Several reasons support the approach based on intrathecal administration: efficient delivery to spinal motor neurons, minimal systemic exposure, reduced peripheral side effects, and sustained therapeutic concentration in the CSF due to slow clearance.

Moreover, the results from clinical trials demonstrated the compelling efficacy of IT-delivered nusinersen. For example, the ENDEAR trial (2016), which included infantile-onset SMA, demonstrated that nusinersen significantly improved motor milestones and survival compared to the sham control [116]. Another trial, CHERISH (2017), included patients with later-onset (Type II) SMA who received nusinersen, and showed significant gains in motor function (HFMSE scores) [117]. The SHINE trial and long-term extensions continued to show durable benefits [118]. Additionally, real-world registries (e.g., SMArtCARE, RESTORE) have confirmed these findings across diverse clinical settings and genotypes. Importantly, early initiation—especially in pre-symptomatic individuals—yields the most profound benefits, aligning with the concept of a therapeutic window before irreversible motor neuron loss [119].

Despite favorable outcomes, IT nusinersen delivery has also brought technical and logistical challenges. Particularly in infants and young children, sedation and specialized expertise are required. Patients with severe scoliosis or spinal fusion, commonly encountered in Type II and III SMA, pose therapeutic challenges, as standard lumbar access may be impossible. Image-guided (fluoroscopic or CT) intrathecal delivery and implantable reservoirs (e.g., subcutaneous intrathecal catheters) could be potential solutions. Furthermore, studies are investigating modified ASOs with enhanced stability and tissue penetration, potentially allowing less frequent dosing or alternative routes (e.g., intraventricular).

SMA is a success story that exemplifies the triumph of precision medicine in neurology. Intrathecal therapeutic administration has been instrumental in offering a meaningful outcome to a once-fatal disease. Nusinersen, delivered intrathecally, has set a precedent for treating genetic CNS disorders at their source. Several factors could explain the contrast between the success of IT therapy in SMA and the more modest or uncertain outcomes in other NDDs. First, SMA is a monogenic disease with a well-defined molecular target (*SMN2* splicing), allowing for precise ASOs design and a clear biomarker (SMN protein) for pharmacodynamic assessment. Second, the disease affects motor neurons throughout the CNS but spares much of the BBB integrity in early stages, enabling relatively homogeneous distribution of IT-delivered ASOs to relevant spinal cord regions. Third, SMA patients, particularly infants and young children, have faster CSF turnover and shorter neuraxial lengths, facilitating more even drug distribution compared to the heterogeneous CSF flow patterns in adult-onset NDDs, such as AD and PD. Finally, clinical trial design benefited from robust natural history data, sensitive functional endpoints, and early intervention in a rapidly progressive disease, all of which enhanced the ability to detect meaningful treatment effects. These lessons underscore that IT delivery is most likely to succeed when the therapeutic target is genetically validated, the distribution requirements match achievable CSF pharmacokinetics, and endpoints are sensitive enough to capture early functional gains. Still, there are challenges that need to be overcome, detailed in the following section, with current IT delivery techniques needing refinement.

## 4. Challenges in Intrathecal Drug Delivery and Potential Alternatives

Intrathecal administration of therapeutics has gained prominence as a viable route for delivering therapeutic agents in neurological diseases, with SMA being the first successful story in clinical practice. Still, besides the incontestable advantages of IT drug delivery, there are anatomical, pharmacological, procedural, and logistical challenges that need to be overcome. Table 4 summarizes the most relevant challenges and potential solutions to improve intrathecal drug delivery.

From an anatomical perspective, the main challenges are related to accessing the intrathecal space, complications arising from repeated dosing, and the biological barriers of the human body. Lumbar puncture is the standard route for IT drug administration but becomes increasingly difficult in NDD patients due to age-related degeneration, comorbidities (e.g., osteoporosis), and disease-specific complications, including severe scoliosis, vertebral collapse, or spinal fusion surgeries [120]. In such cases, fluoroscopy-guided or CT-guided access is necessary, which increases costs, radiation exposure, and procedural time [121].

With most IT-administered therapies requiring chronic administration (e.g., every 4 months for nusinersen), this entails repetitive lumbar punctures, each procedure introducing procedural risks, including post-dural puncture headache, infections, CSF leaks, and decreased patient compliance. Thus, for long-term success, neuroanesthesia support is required.

Disease-specific conditions alter the already highly selective biological barriers, CSF dynamics, immunogenicity, and inflammatory responses. Many NDDs alter the CSF flow and composition: hydrocephalus ex vacuo in AD, spinal canal narrowing in ALS, and chronic inflammation affecting drug absorption and metabolism [126]. Additionally, repeated IT administration of biologics or oligonucleotides can lead to CSF pleocytosis, or more severe conditions such as sterile meningitis or choroid plexus inflammation [127].

Besides anatomical challenges, pharmacological aspects also need to be addressed to improve the outcome of IT-administered therapies. Uneven drug distribution in CSF remains a problem, being influenced by CSF dynamics and pulsatility, and also by molecular weight and charge of the drug [122]. Medication concentrations tend to decline rostrally, leading to subtherapeutic levels in brain regions. This is particularly problematic for diseases like HD or AD, where supraspinal targeting is essential. Another relevant aspect is the limited parenchymal penetration, particularly for large molecules, such as ASOs, which face significant penetration limitations through natural barriers. This results in periventricular or superficial CNS targeting, with limited effect on deeper nuclei [123].

Technological and device-related issues represent another critical limitation of IT drug delivery-based therapies. For chronic administration, implanted catheters or subcutaneous reservoirs (e.g., intrathecal pumps) have been proposed as good alternatives to repetitive lumbar punctures for IT medication administration [36,37]. Still, these devices carry a risk of infection, dislodgement, and fibrosis, while pump systems may malfunction, especially with viscous or particulate-loaded formulations. When considering NDD patients who need periodic imaging monitoring, device-related MRI compatibility and artifact generation remain a concern. Additionally, there is a lack of standardization, with significant variability in drug formulation (concentration), delivery volume, and rate, which results in inconsistent clinical outcomes and complicates regulatory approvals [124].

Even when addressing the abovementioned issues, the healthcare system and the economic burden remain relevant limitations. IT drug administration requires an adequate infrastructure, skilled proceduralists, sterile procedural environments, and long-term follow-up clinics, all of which are found in tertiary centers, making global generalized accessibility a significant concern [128]. The economic burden is generated not only by the cost of the drug, but mainly due to procedural costs, imaging, and monitoring requirements. These costs are magnified in chronic progressive diseases, creating sustainability concerns for health systems and payers [129].

Finally, in particular cases, ethical and psychosocial aspects could become serious limiting factors for IT administration of therapeutics. Special considerations should be taken when treating pediatric patients (special consents). Sedation risks should be more thoroughly explained to the patients. Additionally, the balance between disease progression (late-stage NDDs with associated comorbidities) and procedural benefits should be regularly assessed in chronic administration protocols [125].

Addressing all these factors is highly relevant, as it significantly impacts treatment adherence, improves patients’ quality of life, and ultimately leads to better long-term outcomes.

Ultimately, there are also alternative routes to the lumbar intrathecal administration, to deliver therapeutic agents into the CSF, with intracisternal (cisterna magna) and intraventricular approaches as two notable possibilities that deserve consideration due to their potential advantages in terms of biodistribution and CNS targeting. Several preclinical and translational studies have demonstrated that cisterna magna (CM) injections can achieve broader and more efficient rostral CNS distribution than lumbar puncture, with reduced procedural complexity compared to stereotactic intracerebral approaches. For example, Chen et al. (2023) provided a systematic comparison of CSF delivery routes for AAV-based gene therapy, highlighting significant variability in vector biodistribution between lumbar, intracisternal, and intracerebroventricular routes [130].

Moreover, Hinderer et al. (2018) reported that CM administration of AAV9 in dogs and non-human primates achieved widespread brain and spinal cord transduction, with a lower incidence of inflammatory responses compared to intraventricular injection [131]. In parallel, Taghian et al. (2020) described a minimally invasive CM delivery technique in sheep that demonstrated high CNS bioavailability and is now being translated into clinical protocols [132].

Intraventricular administration, although more invasive, has also been used successfully in various models, particularly for targeting ependymal cells as a source of continuous therapeutic protein secretion, as demonstrated in the work conducted by Yamazaki et al. (2014) [133]. Table 5 summarizes, in a comparative manner, the alternative routes for drug delivery to the CNS.

Collectively, these findings suggest that intracisternal and intraventricular routes may serve as viable or even preferable alternatives to lumbar IT administration for specific therapeutic modalities, especially those requiring widespread cortical or deep brain distribution. Future studies comparing these delivery strategies in terms of pharmacokinetics, biodistribution, safety, immunogenicity, and patient tolerability will be crucial to optimizing CSF-targeted therapies for neurodegenerative diseases.

## 5. Next-Generation Drug Delivery Systems for Intrathecal Therapies

Despite significant improvements in the field, the current drug delivery platforms still remain limited by poor CSF and parenchymal distribution, infection and mechanical complication rates, fixed-rate or manually adjusted dosing, and an inability to clear pathogenic proteins or precisely control biologic exposure over long timescales. Next-generation drug delivery systems must address these gaps, and innovations are needed in all aspects of the devices. Subcutaneous access ports and refined catheter architectures should be optimized to reduce the risk of infection and enable safer, repeated CSF access for bolus or programmable therapy administration. A relevant example is the minimally invasive lumbar port (MILP) system, which was successfully implanted and maintained in animal models by MacAllister et al. [134]. The study describes the development of an MILP model for serial CSF collection in rhesus macaques as an alternative to surgically invasive ventricular reservoir and lumbar laminectomy models. Unlike existing systems, MILP implantation does not require stereotaxic placement, MRI guidance, or extensive postoperative care, and was well tolerated without neurologic deficits, infection, or skin erosion. The model achieved a 70% successful establishment rate over 3 months, with 57% of devices maintaining long-term function (average 19.2 months), reliably yielding 0.5–1.0 mL of CSF in unanesthetized but restrained macaques for pharmacokinetic/pharmacodynamic studies [134]. While complications included port failure and nonpatency, the MILP provides a closed, reliable, and less invasive system for frequent, atraumatic lumbar CSF sampling, serving as a practical alternative to temporary catheterization and, in some studies, to ventricular access when lumbar CSF is an acceptable surrogate [134].

CSF flow platforms and extracorporeal/convective exchange approaches are being developed specifically to homogenize distribution and overcome the stagnation zones that limit lumbar-to-ventricular transport [135]. Next-generation intrathecal systems should center on implantable, programmable infusion pumps and surgically implanted access ports that enable repeat, precise CSF dosing. Starting from currently available pumps such as Medtronic’s SynchroMed line [136], it is clear that there is a need to improve the programmable parameters of bolus infusion via intrathecal catheters. Clinical and bench data should confirm the high delivery accuracy and the ability to tailor dosing over time.

**Figure 6:**
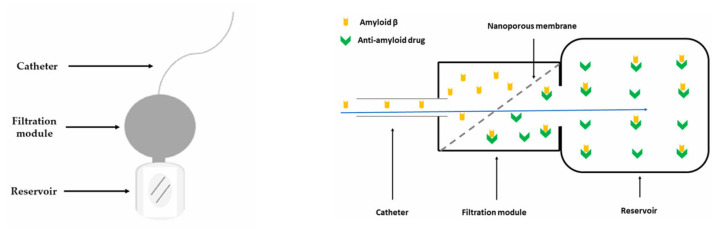
Schematic representation of an experimental CSF filtering device (**left**) and of the filtration module, adapted from Schreiner et al. [137], where the Aβ-rich CSF travels through the nanoporous ceramic filter (blue arrow), the size-based filtration takes place, and subsequently, via a specific antigen–antibody reaction, Aβ is permanently sequestered in the reservoir (**right**).

Concurrently, “nanoporous membrane”-based implantable reservoirs and intrathecal pseudodelivery devices (which present affinity-based sinks or size-selective membranes that permit molecular exchange without systemic exposure) offer a way to capture or slowly release large biologics directly in CSF while minimizing peripheral toxicity. One relevant example is an experimental device capable of filtering Aβ from the CSF developed and tested in an AD mouse model by Schreiner et al. [137]. The authors presented in their paper the multistep process of developing a functionalized biocompatible nanoporous ceramic membrane, followed by a complex evaluation of the membrane’s structural and functional integrity, and finally its use in mice. The nanoporous ceramic filter-based system, which selectively filters Aβ from the CSF, seemed to be a feasible and safe treatment modality in the AD mouse model, with the prototype having a functional lifespan of around four weeks (see Figure 6). With future research focusing on the development of advanced nanoporous ceramic filters with anti-biofouling properties, which ensure long-term therapeutic action, these devices could be promising tools for antibodies and aggregate-clearing strategies.

Simultaneously, the field is moving toward innovative adaptive systems: artificial intelligence (AI)-enabled closed-loop pumps and sensor-guided platforms can tailor intrathecal dosing in real time based on physiological or biomarker feedback, thereby improving efficacy, lowering dose, and diminishing side effects. Initial variants of flow sensors have been tested in recent years, with a particular focus on those that can operate within the clinical range for CSF flow. One example is the pressure-sensitive capacitor sensor developed by Raj et al., which is suitable for long-term implantation and features a wireless external spectrometer for measuring passive subcutaneous components [138]. The study describes the development of an MEMS-based capacitive pressure and flow sensor designed for integration into CSF shunt systems to monitor intracranial pressure and shunt function. The device is fabricated on silicon wafers with carefully engineered capacitor membranes (520 μm wide, 0.5 μm plate spacing) optimized for high sensitivity to the very small pressure and flow ranges relevant to CSF dynamics. The sensor chip is mounted on a biocompatible PMMA carrier with a fluid channel, sealed, and coupled with inductors to enable wireless readout of the resonant frequency. In a “twin capacitor” configuration, the device simultaneously measures flow (via pressure drop between sensors) and intracranial pressure (via averaged signal), with correction for gravitational effects using a tilt sensor. Bench testing demonstrated highly accurate quadratic relationships between frequency and both flow and pressure, with resolutions of ~0.6 mL/h for flow and 0.01 cm H_2_O for pressure, minimal drift, and sensitivity sufficient for clinical application. Compared with existing devices, this sensor offers miniaturization, wireless readout, simultaneous flow and pressure monitoring, and incorporation into standard shunt systems, providing a robust tool for early detection of shunt malfunction or occlusion while minimizing invasiveness [138].

Advancements in “smart” and sustained-release IT platforms, which reduce hardware demands or add responsive control, are mandatory for future improved devices. Bioactive hydrogel scaffolds offer local, sustained release, and trophic support, enhancing tissue retention and regenerative potential. Several research groups have already tested bioactive hydrogels in spinal cord injury models. For example, Sun et al. fabricated composite hydrogels (CRP) based on chitosan, RADA_16_ nanofibers, and nerve-promoted, with excellent injectability, superior biodegradability, and biocompatibility. CRP promote the proliferation and migration of bone marrow mesenchymal stem cells and also induce the proliferation and differentiation of neural stem cells into neurons [139]. In addition, nano- and microparticle carriers delivered intrathecally are being engineered to navigate CSF flow, improve rostral distribution, and modulate pharmacokinetics across the CNS—an area captured in recent comprehensive reviews of IT nanoparticle delivery for CNS disease [140]. The literature indicates that the brain penetration by nanoparticles is influenced by their properties and functionalization. IT delivery of nanoparticles achieves higher and prolonged drug concentrations in the brain, improving distribution and minimizing systemic exposure. Various types, including lipid-based, cell-derived biomimetic, inorganic, and polymeric types, show promise in current studies [140], with liposomal nanoparticles being particularly effective. Engineered nanocarriers, including polymeric nanoparticles, solid lipid nanoparticles, liposomes, micelles, dendrimers, and more advanced systems like nanogels and nanotubes, offer benefits such as enhanced CNS penetration, extended circulation, and sustained release. However, their efficacy depends on critical physicochemical properties (size, shape, charge, biodegradability, and biocompatibility), while concerns remain about solubility, toxicity, clearance, and limited deep parenchymal penetration [141]. Uptake across the blood–cerebrospinal fluid barrier primarily occurs via receptor-mediated transcytosis (RMT), utilizing pathways such as transferrin, insulin, LDL receptors, LDL receptor-related proteins (LRP1, LRP2, LRP8) [142], folate receptors, and plasma protein transport mechanisms, with the choroid plexus epithelium playing a crucial role [143]. Although RMT and novel strategies, such as folate receptor-mediated exosome transport, show great promise, optimizing ligand–receptor interactions, minimizing toxicity, and standardizing safety assessments remain essential for the clinical translation of NP-based CNS drug delivery [144].

Integrating these technologies is crucial for providing a path to safer and more effective intrathecal therapies for neurodegenerative diseases. However, clinical translation will require rigorous comparative pharmacokinetic/pharmacodynamic studies, standardized safety endpoints, and multidisciplinary device–drug regulatory strategies [145].

## 6. Conclusions

With the increasing incidence and prevalence of NDDs worldwide, and yet no curative treatment for general use, IT-administered drug therapies offer a promising avenue in this context. The IT route has several advantages when pharmacologically targeting the CNS, including the ability to bypass the BBB; however, it also poses significant challenges to its adequate implementation. Lumbar puncture, intrathecal catheters, and implantable intrathecal infusion pumps are the primary approaches in IT drug delivery, each with its own strengths and drawbacks.

Considering the most relevant NDDs, IT treatments include mainly ASOs, gene therapy using viral vectors such as AAVs, and small interfering RNAs (siRNAs). With a small number of phase 1 and 2 clinical trials currently in progress, it is difficult to draw definitive conclusions yet. The only medication already approved and with a verified positive impact based on real-world data is nusinersen, used in the treatment of SMA. Still, this success story also demonstrates the multiple anatomical, pharmacological, economic, and sometimes ethical challenges that need to be addressed and overcome before realizing similar IT-administered drugs on the market.

With these great perspectives, there are a couple of directions that need to be addressed in order to improve the outcomes of IT-delivered drugs. Firstly, there is a pressing need to improve delivery techniques and resolve device-related limitations. Secondly, there is a high need for standardization of administration protocols and drug formulations. Finally, the economic burden related to the healthcare system necessitates rethinking the infrastructure to respond to the needs of both the administration and follow-up.

## Figures and Tables

**Table 1:** Advantages and limitations of the most relevant approaches and devices for intrathecal drug delivery.

Device/Approach	Advantages	Limitations	Patient Compliance	CNS Coverage	Relevant References
Lumbar puncture	Low cost Minimally invasive No surgical implantation	Patient discomfort and/or anxiety Post-puncture headache	Low-moderate (reduced adherence when weekly/monthly)	Limited for supratentorial targets	[30,31]
Intrathecal catheters	Controlled drug delivery and dosing Possibility of combination therapies	Risk of infection, catheter dislodgement, or occlusion Routine monitoring	Moderate (requires clinic visits and patient acceptance of the implanted device)	Intermediate (improved cumulative CSF exposure, but heterogenous distribution)	[32,33,34]
Implantable intrathecal infusion pumps	Continuous, steady-state drug delivery	Surgical implantation Pump malfunction, infections, and catheter-related complications	Moderate-high (Once implanted, minimization of repeated procedures and generally improve long-term adherence	Best for maintaining steady CSF levels (sustained gradients and homogeneous CSF concentrations for large molecules)	[35,36,37]

**Table 2:** Current status of clinical trials in AD (↑—increase in value).

Therapeutic Class	Target/Mechanism	Lead Compound/Vector	Trial Phase	Key Findings/Status
ASOs–Tau-targeted	MAPT mRNA degradation via RNase H, leading to decrease in tau protein synthesis	BIIB080 (IONIS-MAPTRx)	Phase 1b (NCT03186989); Phase 2 (NCT04986707)	Phase 1b: Well tolerated up to 60 mg IT q12w; dose-dependent decrease in CSF total and p-tau; sustained effect; exploratory imaging suggests atrophy stabilization. Phase 2 ongoing (long-term efficacy, cognition).
ASOs–APP-targeted	APP mRNA suppression leading to decrease in Aβ42 production	Not yet publicly named	Preclinical/Early clinical	Preclinical data: substantial Aβ42 reduction without major physiological disruption; IT route used for CNS precision delivery; no late-stage trials yet.
Monoclonal antibodies–Anti-Aβ	Direct binding to Aβ to promote clearance	Investigational anti-Aβ mAb	Phase 1 (NCT03397506)	IT administration yields higher CSF-to-plasma ratios vs. IV; potential to lower dose and reduce ARIA; PK results pending; efficacy/engagement endpoints planned in future trials.
Gene Therapy–Neurotrophic factors	NGF/BDNF expression for cholinergic neuron support	IT-AAV-NGF; IT-AAV-BDNF	Preclinical; early clinical (e.g., NCT00087789–stereotactic NGF)	Stereotactic NGF: long-term transgene expression, ↑ ChAT; IT-AAV in animals shows hippocampal/cortical expression and memory improvement; IT-AAV-BDNF Phase 1/2 planned.
Gene Therapy–Amyloid/Tau modulation	Enzymes or RNAi to degrade Aβ or suppress tau	IT-AAV-neprilysin; tau-lowering RNAi AAV	Preclinical	IT delivery reduces plaque/tau pathology, improves cognition; IND-enabling programs ongoing for tau-lowering sequences.
Gene Therapy–Synaptic/metabolic support	Overexpression of PGC-1α, PSD95, Homer1a	IT-AAV vectors	Preclinical	Shown to improve mitochondrial function, reduce oxidative stress, and enhance synaptic resilience in animal models.

**Table 3:** Current status of clinical trials in PD (→—determines the following process; ↑—increase in value).

Therapeutic Class	Target/Mechanism	Lead Compound/Vector	Trial Phase	Key Findings/Status
Gene Therapy–GBA1-targeted	AAV9-mediated delivery of functional *GBA1* gene → ↑ GCase activity → improved lysosomal function and α-synuclein clearance	PR001	Phase 1/2 (PROPEL; NCT04127578); Natural history study (NCT04128245)	Cisterna magna infusion achieves widespread CNS biodistribution; early data: acceptable safety, dose-dependent increase in CSF GCase activity, preliminary decrease in CSF α-synuclein; long-term efficacy data pending.
Gene Therapy–Neurotrophic factors	AAV-mediated delivery of GDNF → activation of GFRα1/RET survival signaling in nigrostriatal neurons	AAV2-GDNF	Phase 1/2 (intracerebral delivery)	Safety and target expression confirmed; limited by slow clinical benefit and delivery constraints; IT/intracisternal approaches under preclinical evaluation for broader distribution.
Protein Delivery–Neurotrophic factors	Direct administration of GDNF protein → neuroprotection and regeneration of dopaminergic neurons	Recombinant GDNF	Phase 1 (open-label); Phase 2 (randomized)	Phase 1: motor improvement and PET dopamine signal increase; Phase 2: failed primary endpoints; delivery method variability suspected; IT route explored preclinically.
Encapsulated Cell Therapy	Implantation of cells engineered to secrete GDNF locally	NTCELL	Early phase clinical	Designed for sustained, localized GDNF release; aims to overcome distribution and dosing limitations of protein therapy.
Synthetic GDNF Mimetics	Small molecules mimicking GDNF, BBB-penetrant, bind GFRα1/RET	Preclinical candidates	Preclinical	Intended to bypass delivery barriers, offering non-invasive GDNF receptor activation.
mRNA-based Neurotrophic Factor Therapy	IT-delivered mRNA encoding GDNF or related factors for in situ production	Preclinical candidates	Preclinical	Potential for adjustable, repeat dosing without permanent genetic modification.

**Table 4:** Challenges and potential solutions to improve intrathecal drug delivery.

Challenges	Details	Potential Solutions	Relevant References
Anatomical	Access to the Intrathecal Space Repeated Dosing Requirements Biological Barriers	Fluoroscopy-guided or CT-guided access Neuroanesthesia support	[120,121]
Pharmacological	Uneven Drug Distribution Limited Parenchymal Penetration	Drug structure and functionality optimization	[122,123]
Device-Related Limitations	Technological Limitations Lack of Standardization	Device design improvement International regulations	[36,37,124]
Logistical	Health System Limitations Economic Burden	Infrastructure improvement	[122,123]
Ethical	Patient consent Benefit versus disease progression	Ethical regulations	[125]

**Table 5:** Comparative overview of the alternative routes for drug delivery to the central nervous system (CNS).

Administration Route	Procedure Characteristics	Biodistribution/CNS Targeting	Advantages	Limitations	Relevant References
Lumbar intrathecal	Injection into the lumbar subarachnoid space via lumbar puncture	Variable rostral distribution	Widely used; relatively safe and clinically established	Limited rostral brain penetration; potential variability in distribution	[130]
Cisterna magna (CM)	Injection into the cisterna magna; minimally invasive techniques	Broader and more efficient rostral CNS distribution compared to lumbar	High CNS bioavailability; less invasive than intracerebral stereotaxy; reduced inflammatory response compared to intraventricular	Requires careful anatomical targeting; potential procedural risks near the brainstem	[131]
Intraventricular	Injection directly into cerebral ventricles (often stereotactic)	Strong targeting of ependymal cells; can serve as a source of therapeutic protein secretion	Adequate for continuous therapeutic protein delivery; direct brain access	More invasive; higher risk of inflammation compared to CM route; technically complex	[133]

## Data Availability

All data are available in the manuscript.

## Abbreviations

The following abbreviations are used in this manuscript:

AAV	Adeno-associated viruses
AD	Alzheimer’s disease
ALS	Amyotrophic lateral sclerosis
ARIA	Amyloid-related imaging abnormalities
Aβ	Amyloid beta
CM	Cisterna magna
CSF	Cerebrospinal fluid
HD	Huntington disease
IT	Intrathecal
NDD	Neurodegenerative disorder
PD	Parkinson’s disease
siRNA	Small interfering RNA
